# The role of culture media on embryonation and subsequent infectivity of *Capillaria obsignata* eggs

**DOI:** 10.1007/s00436-012-3143-z

**Published:** 2012-10-03

**Authors:** K. M. Tiersch, G. Daş, G. v. Samson-Himmelstjerna, M. Gauly

**Affiliations:** 1 Department of Animal Sciences, Georg-August-University Göttingen, Albrecht-Thaer-Weg 3, 37075 Göttingen, Germany; 2 Institute of Parasitology and Tropical Veterinary Medicine, Freie Universität Berlin, Königsweg 67, 14163 Berlin, Germany

## Abstract

This study investigated whether infectivity of *Capillaria obsignata* eggs depends on media culture used for embryonation. Intact female worms were kept in one of following four media: 0.5 % formalin, 2 % formalin, 0.1 % potassium dichromate and 0.1 N sulfuric acid. Embryonation rates of the eggs were quantified either daily in intact females for 16 days, or weekly in disrupted females. Infectivity of the embryonated eggs was tested through an experimental infection of chickens with a single dose of 250 eggs/ bird. The vast majority of the eggs (>82 %) in the first two thirds of the uteri was able to complete embryonation, irrespective of the culture media used for incubation. However, only 32.6 % of total eggs could be harvested after disruption of the intact females. Embryonation rates of the eggs from disrupted worms were different among four culture media, with 0.1 N sulfuric acid resulting in the highest embryonation rate (44.2 %). All the experimentally infected birds harboured mature worms, with varying establishment rates depending on the culture media (*P* < 0.001). Incubation of the eggs in potassium dichromate 0.1 % resulted in a lower (*P* < 0.001) establishment rate (10.2 %) when compared with formalin (70.5 and 47.9 % for concentrations at 0.5 and 2 %, respectively) or with 0.1 N sulfuric acid (57.5 %). It can be concluded that most of the eggs in first two thirds of the uteri in the intact females have the potential to complete embryonation without being influenced by the culture media. However, disruption of the intact females results in lower number of harvestable embryonated eggs, with a considerable variation due to culture media used. With the exception of 0.1 % potassium dichromate, any of the three media, particularly 0.1 N sulfuric acid, can be suggested for embryonation of *C. obsignata* eggs.

## Introduction

*Ascaridia galli*, *Capillaria* spp. and *Heterakis gallinarum* are the most prevalent helminth species in chicken (Permin and Hansen 1998; Permin et al. 1999; Ruff 1999; Kaufmann et al. 2011a). A recent study (Kaufmann et al. 2011b) has shown a high prevalence of helminths in free-range and deep-litter system hens. Kaufmann et al. 2011b) found that 75.3 % of free-range hens were infected with *Capillaria* spp., 88 % with *A. galli* and 98 % with *H. gallinarum*. These results confirm earlier studies showing re-emerging helminth infections in the free-range and deep-litter production systems (Permin et al. 1999). Since the prepatent period of *C. obsignata* is shorter (19–22 days; Levine 1938) than that of *A. galli* (4–8 weeks; Idi et al. 2004; Ramadan and Abou Znada 1991) and *H. gallinarum* (23 days; Daş et al. 2011a), the risk of new and re-infections may be higher in floor husbandry systems where chickens have direct contact to their faeces. Nevertheless, many recent studies focused on exploring factors influencing *A. galli* (Permin et al. 1997a, b; Gauly et al. 2001; Gauly et al. 2007; Dänicke et al. 2009; Daş et al. 2012) and *H. gallinarum* infections (Gauly et al. 2008; Daş et al. 2011b), while less effort has been spent on *Capillaria* infections. This may partly have been due to the lack of knowledge regarding establishment of experimental procedures, i.e. preparation techniques for the infection material and effective infection doses, etc., for *Capillaria* infections.

Among four different *Capillaria* species that reside in the intestinal tract of chicken, *C. obsignata* is the most common one (Friedhoff and Ehlers-Bhodigen 1965). *C. obsignata* lives in the mucous membranes of the small intestines and can cause emaciation, diarrhoea, hemorrhagic enteritis and could lead to death (Wakelin 1965). Following sexual reproduction, females lay un-embryonated eggs. The eggs are shed to the external environment through the faeces and undergo development to the infectious larval stage (L1) within 10–21 days, depending on temperature and humidity. The infective eggs may survive over 1 year depending on the environment (Levine 1936a). Following ingestion of the embryonated eggs by the host larvae hatch in the small intestine and reach maturity within 19 days (Wehr 1939).

As embryonation of nematode eggs is affected by several environmental conditions (Dick et al. 1973; Anderson 1992; Permin et al. 1997a) and shows species-specific characteristics, specific requirements must be determined. Two important factors influencing embryonation and the subsequent infectivity of nematode eggs are culture media and duration of incubation. Culture media prevent putrefaction of the *Capillaria* eggs (Levine 1936b) and inhibit the growth of bacteria and fungi (Luttermoser 1938). As shown in Table 1, different media for the cultivation of *C. obsignata* eggs have been described in earlier studies (Graybill 1924; Levine 1936b; Wehr 1939; Long and Wakelin 1964; Norton and Joyner 1965; Berghen 1966). Similar media were used for the cultivation of *A. galli* eggs (Ikeme 1971; Herd and McNaught 1975; Permin et al. 1997a, 1998) and *H. gallinarum* eggs (Sage et al. 2002; Püllen et al. 2008). Formalin and sulfuric acid are often recommended as suitable culture media for *A. galli* and *H. gallinarum* eggs, whereas they have not been comparably tested for *Capillaria* spp. eggs.

**Table 1:** Culture media used for embryonation of eggs for different nematodes of chicken

Author	Helminths	Media	Temperature	Time
Graybill (1924)	*C. obsignata*	Saline solution	22–25 °C	7 days
Levine (1936b)	*C. obsignata*	2 % K_2_Cr_2_O_7_	np	np
Wehr (1939)	*C. obsignata*	Distilled water	AB	6–8 days
Norton and Joyner (1965)	*C. obsignata*	Distilled water	27 °C	10 days
Berghen (1966)	*C. obsignata*	NaCl	25 °C	10 days
Ikeme (1971)	*A. galli*	0.1 N H_2_SO_4_	25 °C	21 days
Herd and McNaught (1975)	*A. galli*	0.5 % Formalin	np	np
Permin et al. (1997a)	*A. galli*	0.1 N H_2_SO_4_	20 °C	30 days
Permin et al. (1998)	*A. galli*	2 % Formalin	32 °C	28 days
Saunders et al. (2000)	*H. gallinarum*	0.5 % Formalin	20–25 °C	21 days
Püllen et al. (2008)	*H. gallinarum*	0.5 % Formalin	AB	2, 4, 6, 8 weeks
Püllen et al. (2008)	*H. gallinarum*	2 % Formalin	AB	2, 4, 6, 8 weeks
Püllen et al. (2008)	*H. gallinarum*	0.1 % K_2_Cr_2_O_7_	AB	2, 4, 6, 8 weeks
Püllen et al. (2008)	*H. gallinarum*	0.1 N H_2_SO_4_	AB	2, 4, 6, 8 weeks

*AB* ambient temperature (20–22 °C), *np* not provided

For *Capillaria* eggs, it is not clear whether eggs showing higher embryonation rates in a certain medium will also have higher infectivity. Thus any investigation of embryonation of *Capillaria* eggs incubated in different media should ideally be followed by subsequent experimental infection. The aim of this study was to test the impact of various culture media on embryonation rates of *C. obsignata* eggs and their subsequent infectivity in young layer chickens.

## Materials and methods

### *Capillaria obsignata*

*C. obsignata* females collected from the intestines of naturally infected free-ranging laying hens were used for both the examination of incubation conditions on the embryonation of eggs in different culture media and for further experimental infection of chickens. For the worm harvest, the intestines were opened longitudinally and the contents were washed with tap water through a metal sieve with mesh apertures of 100 μm. The remaining contents including *Capillaria* spp. were transferred into Petri dishes to separate *C. obsignata* females under a stereomicroscope as described by Wakelin (1965). All the female worms were placed in a Petri dish as the main pool.

### Media cultures and quantification of embryonated eggs

Formalin (0.5 or 2 %), potassium dichromate (0.1 %) and sulfuric acid (0.1 N) were used as the culture media for embryonation of *C. obsignata* eggs. Two parallel procedures were employed to quantify proportions of potentially embryonable and harvestable embryonated eggs, respectively.

In the first approach, development stages of the eggs within the uteri of intact worms, kept in one of the media were monitored for 16 days. A group of intact female worms (*N* = 20) were randomly selected from the main pool and the total number of eggs located approximately in the first two thirds of uterus (proximal to vulva) of each individual was determined. Average length and width of the eggs in the first two thirds of the uterus were 52.3 ± 3.0 and 36.4 ± 9.9 μm, respectively (*n* = 50 eggs). As the eggs in the last third part of the uterus were small (36.4 ± 9.9 μm in length and 17.2 ± 4.5 μm in width), they were considered immature and thus incapable of embryonation. The intact females were randomly allocated to one of the four media (*n* = 5 per medium). The arithmetic mean number of eggs within the selected part of uterus was similar (*P* > 0.05, one-way analysis of variance (ANOVA)) in worms allocated to one of the four media. For each medium, five marked Petri dishes, each containing a single female, were used. The Petri dishes were kept at room temperature (20°–22 °C). At daily examinations, the embryonation status of the eggs within the uterus was recorded and the percentage of embryonated eggs was determined. Only eggs with an active larva inside were classified as embryonated (Wehr 1939). For each female worm, the days in which the first and last embryonation took place were also recorded.

**Fig. 1:**
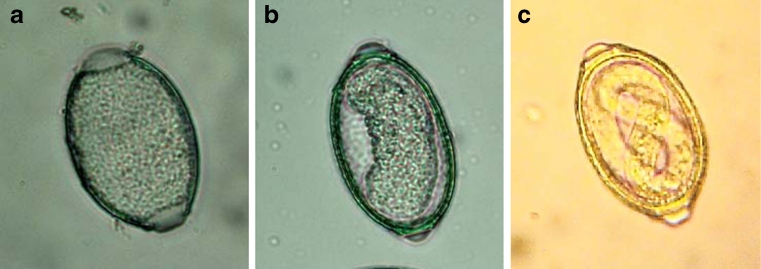
*Capillaria obsignata* eggs in different developmental stages: **a** an un-embryonated egg; **b** a moveable embryo; **c** an infectious larva

In the second approach, 1,000 intact female worms were randomly selected from the main pool and equally allocated to one of the four media (*n* = 250 / medium). For each medium, five Petri dishes, each containing 50 females within 5 ml of culture medium, were used. The Petri dishes were kept at room temperature for 3 weeks as described above. The amount of culture medium in the Petri dishes was kept constant (5 ml). Embryonation rates of the eggs were determined at the end of second and the third weeks of incubation using 25 randomly selected females from each Petri dish (*n* = 500 per week). Selected females were disrupted by using a Potter S Homogenizer containing 1 ml of water in the cylinder and rotated at 800 rpm for three times each 2 s long. Numbers of embryonated and non-embryonated eggs were repeatedly (nine times) determined by counting eggs in sub-samples (10 μl) of the suspension derived from the homogenizer. A presentation of an un-embryonated egg, a moveable embryo and an infectious larva is given in Figure 1.

### Experimental infection

A total of 20 1-day-old male White Leghorn (Lohmann Selected Leghorns) chicks were reared in a parasite-free environment. The birds received water and a commercial grower diet *ad libitum*. At an age of 3 weeks, the birds were numbered with wing tags, weighed and allocated to one of four groups based on the culture media used for embryonation of the eggs to be used for the infection. Each group (*n* = 5) was kept in a separated growth unit, and the individual birds were orally infected with a single dose of 250 embryonated eggs of *C. obsignata*, which were cultivated in one of the four corresponding media for 3 weeks as described above. The oral infection was performed using a 6-cm-long buttoned cannula, which was placed into the crop on day  0, i.e. at an age of 3 weeks. The birds were weighed on the infection day and 4 weeks after infection. On days 17, 19, 23 and 28 post-infection (p.i.), the birds were placed into single cages for collecting individual faecal samples. Faecal egg counts were performed using a modified McMaster technique (MAFF 1986). The study design did not include an uninfected control group of birds. Post-mortem examinations were performed on day 28 p.i. and the intestines were removed and all *Capillaria* were collected, counted, differentiated for sex and worm length was quantified as described by Wehr (1939).

### Statistics

Embryonation rate of the eggs within first two thirds of uterus was calculated as the proportion of larva-containing eggs to the number of eggs counted. Embryonation of the eggs within a female was evaluated in two time periods. The first period was defined as a lag period, the time required until an egg fully developed into a larva (first embryonation). The second period was defined as the time at which the last embryonation took place in a female worm (last embryonation). Differences among media for embryonation rate, for the time required for the first and last embryonations were analysed with one-way ANOVA.

Data regarding proportions of embryonated, un-embryonated and damaged eggs from the worms kept in different media and disrupted shortly before counting were analysed with a two-way ANOVA model that included effects of media, incubation weeks and the interaction between these two factors. Worm counts, worm length, body weight on the infection day and average daily weight gain during the infection period (4 weeks) were analysed with one-way ANOVA, considering the media effect.

Faecal egg counts (eggs per gramme faeces, EpG) were log transformed with a function [*y* = log10 (*y* + 10)] to get approximately normally distributed data. Log transformed EpG data were analysed with repeated measures ANOVA using proc Mixed of SAS. The model included the fixed effects of media, sampling days (19, 23, 28 d.p.i.) and the interaction effect between media and sampling days. The effect of repeatedly sampled bird (subject) was included in the model as random. As there was no egg (EpG = 0) in any of faeces samples on day 17 p.i., data obtained from this day were excluded from the analyses. All post hoc comparisons were performed with Tukey test with a significance level of *P* < 0.05. The statistical analyses were performed with SAS (2010).

## Results

### Embryonation potential of eggs within the uteri of intact females

**Fig. 2:**
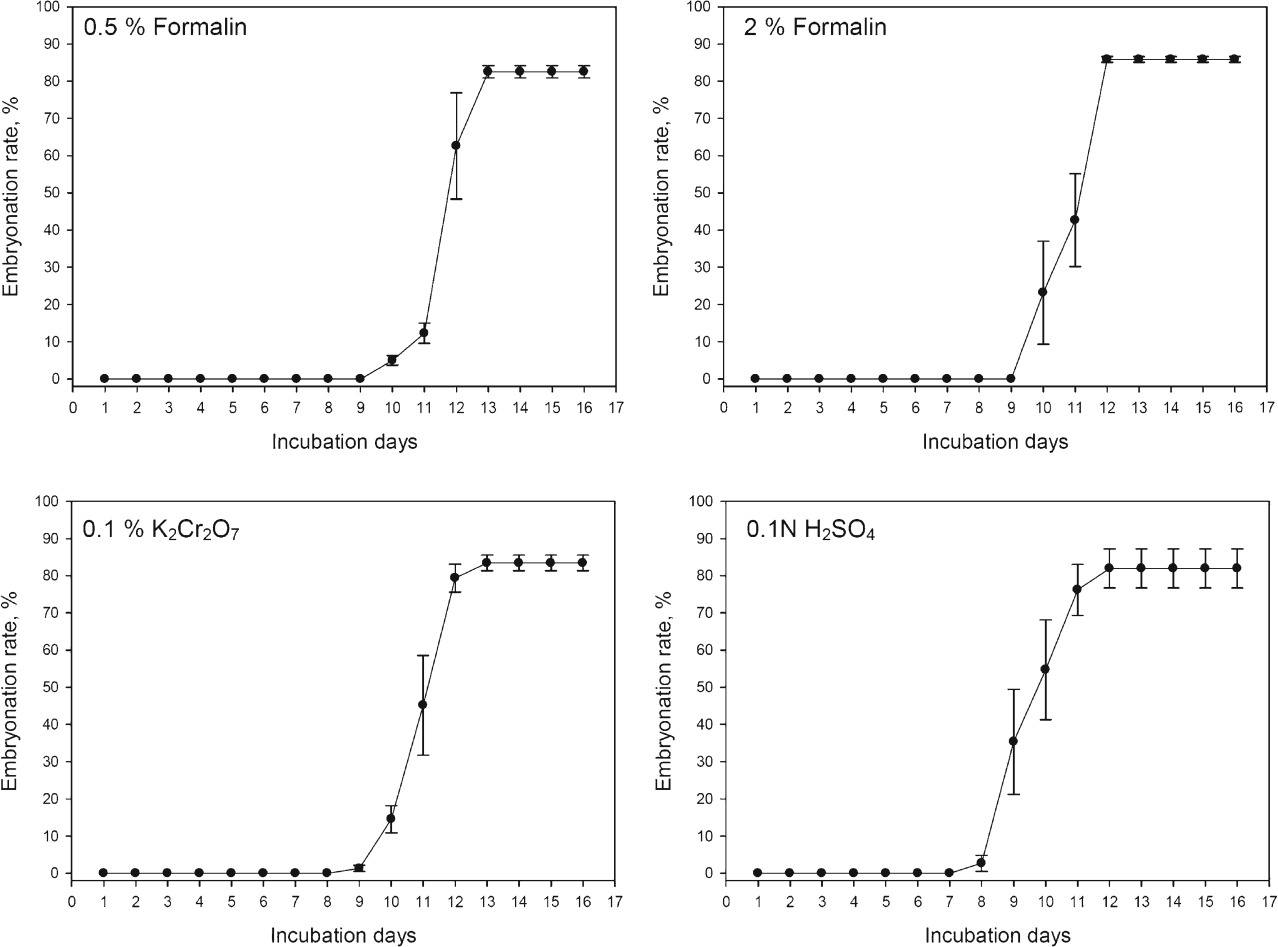
Embryonation of the eggs in the first two thirds of the uteri of intact female worms kept in different culture media (means and SE on the *error bars*)

Most of the eggs (>82 %) in the first two thirds of the uteri were embryonable (Fig. 2), whereas none of the eggs in the last third of the uteri fully embryonated during the 16 days of observations. In all media, all the embryonable eggs in the first two thirds of uterus accomplished embryonation fully latest around day 13. As shown in Fig. 2, completion of embryonation in all the eggs within uterus took place within 2–3 days after the first egg had fully embryonated. After 16 days of incubation, there was no significant effect of culture media on the embryonation rate of the eggs in the uteri of the intact female worms (*P* = 0.807; Table 2). The embryonation rates of the eggs in the first two thirds of the uteri ranged from 82.0 % (SE = 0.8) to 85.8 % (SE = 5.3) among four different media. The time until first embryonation occurred was significantly (*P* < 0.001) shorter for the worms incubated in 0.1 N sulfuric acid when compared to other three media. Similarly, the time required until the last embryonable egg completed embryonation tended (*P* = 0.052) to be shorter for worms incubated in 0.1 N sulfuric acid media.

**Table 2:** Embryonation rate, the time required until the first and last embryonation took place within intact female worms and the average proportions of harvested eggs (embryonated, un-embryonated and broken–damaged) from female worms kept in different media during incubation and disrupted shortly before counting (means ± SE)

	0.5 % Formalin	2 % Formalin	0.1 % K_2_Cr_2_O_7_	0.1 N H_2_SO_4_	*P*≤
In intact females					
Embryonation rate, %	82.5 ± 1.69	85.8 ± 0.79	83.4 ± 2.1	82.0 ± 5.30	0.807
First embryonation day	10.2 ± 0.20a	10.2 ± 0.20a	9.6 ± 0.24a	8.6 ± 0.24b	0.001
Last embryonation day	12.4 ± 0.24	12.0 ± 0	12.4 ± 0.24	11.4 ± 0.40	0.052
Harvested eggs					
Embryonated, %	24.0 ± 2.67a	28.9 ± 3.48a	33.1 ± 2.48ab	44.2 ± 3.35b	0.001
Un-embryonated, %	48.4 ± 4.91a	42.3 ± 4.56ab	38.9 ± 3.49ab	27.5 ± 2.87b	0.008
Broken–damaged, %	27.6 ± 6.82	28.8 ± 5.55	28.0 ± 3.89	28.3 ± 3.36	0.999

Different letters (a and b) indicate significant differences between the media (Tukey, *P* < 0.05 after a significant media effect, *P* < 0.01)

### Embryonation rates of the eggs harvested from disrupted females

Overall average proportions of embryonated, un-embryonated and damaged eggs harvested from the intact worms that were kept in different media and disrupted shortly before counting, were 32.6 % (SE = 1.8), 39.3 % (SE = 2.3) and 28.2 % (SE = 2.4), respectively. There were no significant (*P* > 0.05) interaction effects between media and incubation weeks on the proportions of embryonated, un-embryonated and damaged eggs. Similarly, no significant (*P* > 0.05) main effect of incubation weeks was observed on the proportions of embryonated, un-embryonated and damaged eggs. Culture media had significant effects on the proportions of embryonated and un-embryonated eggs from the disrupted worms (Table 2; *P* < 0.01). In comparison to formalin (0.5 and 2 %), 0.1 N sulfuric acid increased (*P* < 0.05) the proportion of harvested embryonated eggs. Accordingly, the proportion of un-embryonated eggs was higher in the eggs incubated in formalin 0.5 % when compared to sulfuric acid. Proportions of embryonated as well as un-embryonated eggs incubated in formalin (0.5 % or 2 %) and potassium dichromate did not differ significantly (*P* > 0.05).

### Experimental infection

**Fig. 3:**
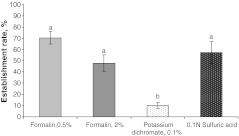
Establishment rates of *C. obsignata* eggs incubated in different culture media and given to chickens with single dose of 250 eggs/bird (means and SE on the *error bars*). *a, b different letters* indicate significant differences between the media (Tukey, *P* < 0.05 after a significant media effect, *P* < 0.001)

None of the birds died, and noticeably no clinical sign of infection was observed during the infection period (4 weeks). All the experimentally infected birds harboured mature worms, with varying worm establishment rates depending on the incubation media (*P* < 0.001). As shown in Fig. 3 incubation of the eggs in potassium dichromate 0.1 % resulted in a lower (10.2 %) establishment rate when compared with either concentration of formalin (70.5 and 47.9 % for concentrations of 0.5 and 2 %, respectively ) or with 0.1 N sulfuric acid (57.5 %). There was no significant difference between formalin and sulfuric acid media in their effects on the worm establishment rates (*P* > 0.05). Correspondingly, the mean numbers of female and male worms as well as total worm burdens were not different (Table 3; *P* > 0.05) between formalin (0.5 and 2 %) and sulfuric acid, whereas incubation of eggs in potassium dichromate significantly lowered worm counts (*P* < 0.001). There was no significant effect of culture media on worm length (*P* > 0.05).

**Table 3:** Mean values (±SE) for parasitological parameters and ADG of the chickens infected with 250 infectious *C. obsignata* eggs incubated in different culture media

	0.5 % Formalin	2 % Formalin	0.1 % K_2_Cr_2_O_7_	0.1 N H_2_SO_4_	*P*≤
Worm burden, *n*	176.2 ± 14.50a	119.8 ± 18.34a	25.4 ± 7.10b	143.8 ± 24.86a	0.001
Females, *n*	74.2 ± 6.94a	74.0 ± 11.85a	11.6 ± 4.47b	77.2 ± 9.32a	0.001
Males, *n*	102 ± 9.37a	45.8 ± 8.53a	13.8 ± 2.70b	66.6 ± 16.10a	0.001
Larvae, *n*	1.8 ± 0.49	1.2 ± 0.20	0.80 ± 0.49	3.0 ± 1.67	0.375
Female length, mm	14.4 ± 0.82	14.5 ± 0.34	15.7 ± 0.68	13.5 ± 0.17	0.133
Male length, mm	10.3 ± 0.71	10.6 ± 0.15	11.5 ± 0.58	10.35 ± 0.27	0.308
Average EpG^a^	161 ± 37a	126 ± 46ab	30 ± 15b	148 ± 31a	0.005
ADG^b^	14.2 ± 0.34	14.9 ± 0.45	16.0 ± 0.15	12.9 ± 2.17	0.330

Different letters (a and b) indicate significant differences between the media (Tukey, *P* < 0.05 after a significant media effect, *P* < 0.001)

^a^Values represent untransformed data, *P* values and multiple comparisons are based on transformed data

^b^ *ADG* average daily weight gain

Faecal egg counts of the birds (EpG) were significantly influenced by both culture media (Table 3; *P* = 0.005) and sampling days (*P* < 0.002), whereas there was no significant interaction effect between culture media and sampling days (*P* > 0.05). As indicated in Table 3, overall average EpG was lower (*P* = 0.005) in birds infected with the eggs cultivated in potassium dichromate than in the birds infected with the eggs cultivated either in formalin 0.5 % or in sulfuric acid. Overall average EpG counts in birds infected with the eggs cultivated in formalin 2 % tended (*P* = 0.097) to be higher when compared to the EpG counts of birds infected with the eggs cultivated in potassium dichromate. In all the birds, EpG counts gradually increased from days 19 to 28 p.i. (data not shown).

Body weights of the birds were similar on the infection day (*P* > 0.05). In the end of infection period (day 28 p.i.) there was no significant difference among average daily weight gain (ADG) of the birds infected with eggs cultivated in different media (Table 3; *P* = 0.330).

## Discussion

The vast majority of the eggs (82–86 %) in the first two thirds of the uteri of the intact females were able to embryonate within 2 weeks, irrespective of the culture media used for incubation. Eggs in this localization of the uterus can be considered as fully developed and capable of embryonation, since their size correspond well to those found in freshly deposited faeces (Graybill 1924; Wehr 1939). Accordingly, Norton and Joyner (1965) reported high embryonation rates (95 %) for *C. obsignata* eggs isolated from faeces and incubated in distilled water. However, the average proportion of harvested embryonated eggs after disruption of the intact females was considerably low (32.6 %), and dependent on the culture media used for incubation. The average proportion of harvested embryonated eggs after disruption of the worms ranged from 24.0 to 44.2 % with formalin (0.5 %) and 0.1 N sulfuric acid resulting in the lowest and the highest embryonation rates, respectively. The low average proportions of harvested embryonated eggs do not only result from the involvement of immature eggs in the calculations, but also due to the mechanical damage done to the eggs during the disruption of the worms. Although a considerable number of the eggs were damaged (28.2 %) after disruption of intact worms, there was no significant (*P* > 0.05) difference among proportions of the damaged eggs derived from the worms kept in different media. It was not possible to determine the embryonation status of the damaged eggs. Accordingly, similar proportions of the damaged eggs among the four media do not necessarily indicate that disruption of the worms resulted in similar degree of damage to the embryonated eggs of the worms that were kept in different media. In contrast, as the embryonation rates of the eggs in the uteri of intact worms incubated in different media did not differ significantly, it can be inferred that the proportion of embryonated eggs that were damaged during the disruption process was higher for the eggs derived from the worms kept in formalin (0.5 and 2 %) when compared to those kept in sulfuric acid. These results suggest that incubating worms in formalin may have an adverse effect on the integrity of the eggs when compared with sulfuric acid. Effects of formalin and sulfuric acid on embryonation seem to be reproducible for *A. galli* eggs as well. Permin et al. (1997a) isolated *A. galli* eggs from the uteri and incubated either in 2 % formalin or in 0.1 N sulfuric acid, which in turn resulted in embryonation rates of 26 and 41 %, respectively. Since it cannot be ensured that a medium favouring embryonation rates will also increase infectivity of the eggs, embryonation rate alone should not be considered as the main criteria for comparing different media. However, it may rather be used as a parameter to assess the efficiency of a medium for the proportion of embryonated eggs at harvest. This is particularly important when the number of available eggs for embryonation procedures is limited, e.g. in case of rare parasites. However, irrespective of the efficiency of a medium, infection doses are based on the number of infective eggs, and thus infectivity of the eggs should eventually be considered. Infectivity of the eggs, as quantified by the establishment rates, was lowest for the eggs incubated in potassium dichromate, while there was no significant difference among any of the other three media. The impaired ability of the larvae to establish themselves in the intestines might have resulted from toxicity of potassium dichromate solution, which might have induced microscopically non-visible effects on the development of larva inside eggs during the incubation period leading to an impairment of the survival of hatched larva. Potassium dichromate is known as a strong algal cell pollutant (Labra et al. 2007) and chromate salts are corrosive and produce cellular damage to tissue (Anonymous 2010). Burden and Hammet (1976) showed that *Trichuris suis* eggs, which were embryonated in potassium dichromate, had a lower establishment rate (2 %) when compared to eggs embryonated in a control media (20 %). As suggested by Fairbairn (1960) the low infectivity of embryonated eggs may be explained by chemical changes in the egg shell during the embryonation. Kopper and Mansfield (2010) determined that treatment of *Trichuris muris* eggs with 6.25 % hypochlorite-eliminated bacterial and fungal growth on these media, but increased time of exposure to the hypochlorite resulted in increased levels of *T. muris* shell degradation. They suggested that 5- and 10-min exposures of eggs to 6.25 % hypochlorite limited detrimental effects on the integrity of viable egg shells. Application of 6.25 % hypochlorite can be considered as a treatment rather an incubation medium, and it shows that exposure to chemicals for longer time can have an impact on the egg shell. The egg wall of nematode eggs consists of the lipid layer, the chitin and the protein cyst (Eckert et al. 2005). In the present study, the aggressive solution might have affected egg shell layers which in turn resulted in an early hatch and a consequent digestion of larva in the gastrointestinal tract. Although there was no visible change in the morphology of *C. obsignata* eggs incubated in potassium dichromate, size and appearance of the eggs can pronouncedly be influenced under exposure of chemical stressors, e.g. benzimidazoles (Dorny et al. 1987).

With the exception of potassium dichromate, establishment rates of the eggs are comparable to the previous studies. Norton and Joyner (1965) reported 87.5 and 41.3 % establishment rates for *C. obsignata* eggs inoculated with infection doses of 200 and 1,000 eggs/bird, respectively. Despite the significant differences in worm burden of the birds infected with eggs incubated in different media, there were no significant differences for the ADG of the birds. This may partly be explained by the low infection doses. As reported by Norton and Joyner (1965) the most severe symptoms of *Capillaria* infections are observed in birds infected with high doses of eggs (5,000 to 100,000). Since the study design did not include uninfected control birds, lack of differences for ADG of the birds does not necessarily indicate that the infection had no effect on host performance; rather it may indicate that the degree of impairment in growth might have been at similar levels, due to relatively low infection dose. To ensure a sufficient level of experimental *Capillaria* infection with adverse effects on host animal performance, further studies including higher infection doses and uninfected controls in their designs are needed.

## Conclusion

The results showed that most of the eggs in first two thirds of the uteri in the intact females have the potential for embryonation without being influenced by the incubation media. However, disruption of the intact females results in lower number of harvestable embryonated eggs with a considerable variation due to culture media used. With the exception of 0.1 % potassium dichromate, any of the three media, particularly 0.1 N sulfuric acid, can be suggested for embryonation of *C. obsignata* eggs.
